# Managing ancillary care in resource-constrained settings: Dilemmas faced by frontline HIV prevention researchers in a rural area in South Africa

**DOI:** 10.1093/inthealth/ihaa045

**Published:** 2020-11-07

**Authors:** Busisiwe Nkosi, Janet Seeley, Natsayi Chimbindi, Thembelihle Zuma, Maureen Kelley, Maryam Shahmanesh

**Affiliations:** Africa Health Research Institute, KwaZulu-Natal, South Africa; Institute for Global Health, University College London, London, UK; Africa Health Research Institute, KwaZulu-Natal, South Africa; London School of Hygiene and Tropical Medicine, UK; Africa Health Research Institute, KwaZulu-Natal, South Africa; Institute for Global Health, University College London, London, UK; Africa Health Research Institute, KwaZulu-Natal, South Africa; Institute for Global Health, University College London, London, UK; Ethox Centre and Wellcome Centre for Ethics & Humanities, Nuffield Department of Population Health, University of Oxford, UK; Africa Health Research Institute, KwaZulu-Natal, South Africa; Institute for Global Health, University College London, London, UK

**Keywords:** ancillary care, referral process, South Africa, vulnerability

## Abstract

**Background:**

We describe the findings from a research ethics case study, linked with a team evaluating a package of intervention services to prevent HIV infection in adolescent girls and young women (AGYW) living in a rural and poor setting of KwaZulu-Natal, South Africa.

**Methods:**

We conducted qualitative interviews (n=77) with members of the linked research team evaluating the intervention programme, programme implementing staff, AGYW enrolled in the intervention programme, caregivers, ethics committee members, Public Engagement officers, community advisory board members and community stakeholders. Data were analysed iteratively using thematic framework analysis. Themes were determined by the study aims combined with an inductive development of codes emerging from the data.

**Results:**

The findings show that the burden of providing ancillary care fell primarily on the shoulders of frontline researchers and programme staff. Dilemmas around responding to gender-based violence illustrated the limits of ‘referral to services’ as a solution for meeting ancillary care obligations in contexts with barriers to basic health and social services.

**Conclusion:**

Our findings show important gaps in meeting ancillary care needs. Participants’ needs required social and economic support which frontline researchers and implementing partners were not able to meet, causing moral distress.

## Introduction

One of the most persistent ethical challenges in research with participants living with complex health, social and economic needs is determining the scope of researchers’ obligations to respond and what care to provide. The ancillary care provided in health research projects has been defined as: ‘medical care that research subjects need but that is not required to make a study scientifically valid, to ensure a study's safety, or to redress research injuries’^1^ (p.3). A common strategy for addressing such health care needs is to plan for referrals to care and services within the hospital or health system where the research occurs. Yet, as Merritt and colleagues^2^ note, a researcher's responsibilities to facilitate ancillary care ‘through *referral* also raise ethical issues’ (p.105), particularly in resource-constrained settings.^3^^,^^4^ A review of the institutional guidance available in 2010–2012 on ancillary care in low-resource settings, carried out by Krubiner et al.,^5^ found that 23 institutions (half of those they studied) explicitly took a position on the responsibility of researchers to provide ancillary care; the other half recommended that ancillary care be provided through the local health care services. The Council for International Organisations of Medical Sciences (CIOMS) guidelines^6^ recommend that research conducted in low- and middle-income countries (LMICs) ‘make adequate provisions for addressing participants’ health needs during research’ either through the local health system or nongovernmental organisations (Guideline 6, p. 22). However, the nature and scope of ancillary care obligations remain largely unspecified, leaving researchers and research institutions without explicit direction, particularly in LMICs where needs are extensive and services limited; a void that potentially shifts decisions around ancillary care provisions to frontline researchers.^7^ Empirical research ethics studies in LMICs are urgently needed to develop ethical guidance that is more responsive to this context, including addressing the challenges of complex needs beyond health care.

We describe the findings from a research ethics case study, linked with a study team evaluating a package of intervention services to prevent HIV infection in adolescent girls and young women (AGYW) living in a rural and poor setting of KwaZulu-Natal, South Africa. For the case study, we mapped the ethical dilemmas arising in research. For this paper, we focus on the range of ancillary care needs that arose during the evaluation of the intervention. We describe how research staff and intervention implementing partners responded to these needs, the challenges they faced in responding and the insights they shared for improving ancillary care planning in LMICs.

## Methods

### The case study setting

The study was conducted in the Population Intervention Platform Surveillance Area (PIPSA) of the Africa Health Research Institute (AHRI), KwaZulu-Natal, South Africa.^8^ The district of uMkhanyakude, where the PIPSA is based, has a population of 689,090, and is one of the poorest districts in South Africa.^8^ Only about 22% of the population has access to safe water, and just 10% of households are within 15 minutes’ driving time to a health clinic.^9^ Most households depend on small-scale agriculture, state grants and remittances from migrant workers.^10^ Although South Africa is a democratic country, the district is dominated by traditional and tribal structures which inform and shape the local value systems and norms. This dual system – political democracy and traditional governance systems – can at times be a source of tension in determining matters of right and wrong, and how the communities deal with social needs and navigate power dynamics including gender-based violence.

The health system in the District includes five hospitals and 51 primary health care (PHC) clinics.^11^ Most hospital doctors are generalists, and the PHC clinics are nurse led. PHC clinics are visited twice monthly by medical and paramedical staff. Mobile clinics visit some of the more inaccessible areas. The PHC clinics are also supported by a cadre of community care givers, who are based in the communities. Those seeking medical specialists often must travel to the nearest city, approximately 400 km away. Community members enrolled in the AHRI's research often access health care through referrals to local PHC clinics and may be referred on to a district hospital.

The Public Engagement (PE) Department supports the research conducted by the AHRI by enhancing the AHRI's ability to have constructive, interactive and integrated engagement with local communities. The community advisory board (CAB) are members of the community selected by the political and traditional leaders, and the community they represent. CAB members serve a five-year term. They meet every month where they are presented with upcoming studies and provide independent advice.

The intervention project, in which the case study was embedded, was developed to target multiple social, behavioural and biological vulnerabilities to HIV amongst AGYW. These included low employment and educational levels, risky behaviours including alcohol and substance abuse – which exacerbate risks to HIV infection – and unplanned early parenthood.^12^^-^^14^ The intervention project aimed to mitigate the drivers of HIV infection and reduce HIV incidence by 40% among AGYW through civil society partners implementing a combination of biological, behavioural and social services, with beneficiaries expected to access packages of services.^15^^,^^16^ The linked study team were evaluating a large multi-country combination of HIV prevention interventions for AGYW implemented by multiple non-governmental and community-based organisations. These services included HIV testing and counselling, post-violence care (PVC) for survivors of gender-based violence (GBV), community-based interventions to reduce GBV, school-based interventions, financial literacy, condom promotion, parenting programmes and social-asset building. Interventions were implemented in schools, in the community and in health facilities. (See Supplementary Figure 1: Framework for the HIV intervention core package).

Because the intervention study was offering a range of services beyond a narrow biomedical intervention and evaluating these within a resource-constrained health system, it provided a valuable opportunity to understand the ethical realities of responding to complex health and social needs through research.

### Sampling and data collection

The sampling framework was designed to gain insights from the research ecosystem that surrounded the study. We therefore included three cohorts: 1) participants from the research case study (this included researchers, intervention Implementing Partners, AGYW and caregivers, who provided insights and experiences from the researcher and research participant's perspective; 2) ethics committee, PE officers and CAB members, who provided insights into conducting research with vulnerable populations and into ethical dilemmas in research; and 3) community stakeholders, who provided insights into the broader community context and wider perspectives about research in their community. Only AGYW and adolescent boys and young men (ABYM) were recruited, based on age and gender pre-set during the HIV intervention programme. Participants were recruited from rural and peri-urban areas. We did not ask participants for specific ages, but categorised them according to age ranges. We recruited 77 participants. There were five refusals: reasons given were time constraints (n=3) and lack of interest (n=2).

Group discussions, in-depth interviews and key informant interviews were conducted using semi-structured guides (see Supplementary file: Interview guides). Audio-taped interviews lasted from 45 to 60 minutes and were conducted in isiZulu and English by the first author and two research assistants trained in qualitative research methods. We obtained informed consent from participants (aged 18 years and above), and assent from young people aged 10 to 14 years old after parental/guardian consent for the child to participate. We did not use incentives for participation in the study; however, participants were reimbursed for transportation, and refreshments were provided during the interviews. Data collection was conducted between December 2017 and July 2018.

### Data analysis

Transcribed interviews were translated into English and analysed iteratively for themes using thematic framework analysis.^17^ Themes were determined by the study aims and content of the interview guides combined with an inductive development of codes as they emerged from the data. (See Supplementary Table 2: Thematic categorisation and supporting quotes). We conducted initial open coding independently. The study team met regularly to reflect on and revise emerging codes and themes throughout the analysis process. A descriptive narrative approach is used to present the findings, along with participants’ quotes to support and illustrate these findings.

### Regulatory approvals

The research ethics case study was approved by the Biomedical Research Ethics Committee of the University of KwaZulu-Natal (approval number: BE524/17) and the Oxford Tropical Research Ethics Committee (OxTREC), University of Oxford, United Kingdom (approval number: 537–17).

## Results

We describe the experiences of the intervention project and associated research shared by the AGYW, caregivers and community stakeholders, research staff, CAB members and implementing partners, focusing on ethical obligations to respond to health and social needs encountered within research. There were 77 participants, representing three cohorts; see Table 1.

**Table 1.** Data collection methods and study sample

Participants	Age (years)	Data collection method	Sample (n=)
**Group 1: Participants from the intervention-linked study**			
Adolescent girls and young women	10–14	Focus group discussion	6
Adolescent girls and young women	18–24	Focus group discussion	7
Adolescent girls and young women	18–24	Focus group discussion	7
Caregivers	30–40	Focus group discussion	4
Implementing partners	30–40	In-depth interviews	5
Frontline researchers	20–30	Focus group discussion	8
Researchers	30–40	In-depth interviews	2
**Group 2: Ethics committee members, Public Engagement officers, Community Advisory Board members**			
Ethics committee members	50–60	Key informant interviews	3
Public Engagement officers	30–50	Focus group discussion	6
Community Advisory Board members	20–50	Focus group discussion	5
Community Advisory Board members	20–50	Focus group discussion	6
Community Advisory Board members	20–50	Focus group discussion	6
**Group 3: Community members and stakeholders**			
Community members	40–60	In-depth interviews	2
Adolescent boys and men	10–14	Group discussion	3
Adolescent boys and young men	18–24	Group discussion	2
Community caregivers	30–40	Focus group discussion	5
Total:			77

We report on four themes: 1) meeting the participants’ needs in and through research; 2) referral services for gender-based violence and child sexual abuse: 3) how researchers responded to participants’ unmet needs; and 4) lessons for responding to structural care needs within research. Table 2 shows thematic categorisation and indicative quotes.

**Table 2.** Thematic categorisation and supporting quotes

Thematic categorisation	Supporting quotes
Meeting the participants’ needs in and through research	*The community expects us to respond to their needs and we cannot do that. The community needs a lot, they need water, they need housing, roads, education for their children, they need social grants, and they expect this from us*.
	FGD, Public Engagement officer, 30–40 years
	*Some of the members in the community ask me, ‘what does [the institute] help us with, because we do not have a dam for irrigation? They say we must eat healthy food like vegetables when we take pills’.*
	FGD, Community Advisory Board member, female, 30–40 years
Referral services for gender-based violence and child sexual abuse	*The guidelines say if you have been raped, make sure that you get into clinic or you go to the law enforcement within 72 hours. Then when you get there, they [victims] become uncomfortable waiting in the queue, and everyone is looking at them saying ‘maybe she has been raped’*.
	IDI, implementing partner, female, 30–40
	*There are lot of teenagers who have been raped and they never get any assistance. These teenagers would have to travel about 60 km to reach PVC centres. Where is this teenager going to get the money to get assistance?*
	FGD, Public Engagement officer, female, 30–40 years
	*We see very worrying acts and we think ‘you will report [this case], then what will happen next?’*.
	FGD, frontline researcher, male, 20–30 years
How researchers responded to participants’ unmet needs	*But what can you do? You cannot just ignore people's plight like that*.
	Frontline researcher, female, 20–30 years
	…*those things leave me a bit helpless and wish I could do more; […] when the staff come back from the field and they narrate all these sad stories*.
	IDI, investigator, 30–40 years
	*The community looks at us as people who are interested in them only when we want what we want; there's nothing we bring back to people as a form of support*.
	FGD, frontline researcher, male 20–30 years
	*People share their stories and you wonder where do I even begin to help them? What makes it unbearable is the inability to go an extra mile to help them. It makes you emotional.*
	FGD, PE officer, male, 40–50 years
Lessons for responding to structural care needs within research	*Why can't the institute allocate funds which will be dedicated to young people's issues we have raised, such as children who wish to further their studies? That would be a long-term investment in the communities we are working in.*
	FGD, frontline researcher, male, 20–30 years
	*These [vouchers] are not sustainable. What if they give something that can be used long term? …The families are experiencing severe food insecurity.*
	FGD, caregiver, female 30–40 years

### Meeting the participants’ needs in and through research

Dilemmas around the appropriate response to participants’ needs in research were shaped by underlying expectations in the community about how research should respond to participant and community needs. Most AGYW 10–14 and ABYM identified a lack of basic needs, especially food and water, as one of the main challenges in the area. Living within the community where the research took place, the PE officers and CAB members described taking on multiple roles and identities, and saw themselves as ‘advocates’ for the broader community. They described their roles as ‘providing the bridge between research and the community’. They described feeling overwhelmed by the requests they received from the participants in the AHRI studies; requests which were often related to basic services:

*The community expects us to respond to their needs and we cannot do that. The community needs a lot, they need water, they need housing, roads, education for their children, they need social grants, and they expect this from us* (FGD, PE officer, male 20–30 years).

*Some of the members in the community ask me, ‘what does [the institute] help us with, because we do not have a dam for irrigation* *?* *They say we must eat healthy food like vegetables when we take pills* *’* (FGD, CAB member, female, 30–40 years).

Linked study team members explained that while they followed ethical guidelines for addressing participants’ needs during research, many things were beyond the remit of the AHRI to support. To try to address these needs, guidance was in place for referrals to government institutions to facilitate access to services identified during research. The commonest referrals described were for social and welfare services to mitigate food insecurity and facilitate access to social grants. However, these services were overwhelmed and faced backlogs. Impatience from the study participants about the slowness and unresponsiveness of the government processes presented a challenge to frontline researchers who were not in a position to intervene or speed up the processes. Material needs were not, however, the only support which frontline staff identified; addressing GBV, while being one area for intervention in the project package, was particularly challenging.

### Referral services for gender-based violence and child sexual abuse

Violence against women and children is a serious problem in the surrounding communities. Although GBV support services were available in the study setting, there were several constraints which inhibited the level and reach of support offered. For example, PVC sites provide multiple services, including safe spaces, HIV counselling and testing, psychosocial support and legal advice. Yet PVC services were limited to select health facilities collaborating with the intervention programme, so access to these services depended on proximity to those facilities. Therefore, those needing PVC services may have to travel long distances to reach health facilities and access support from law enforcement.

In addition to barriers to access, there is also lack of trust in the services. Intervention implementing partners reported that the services were often not responsive and lacked private space, so most participants were reluctant to seek assistance, even when they had been made aware of the support. An implementing partner staff member commented:

*The guidelines say if you have been raped, make sure that you get into clinic or you go to the law enforcement within 72 hours. Then when you get there, they [victims] become uncomfortable waiting in the queue, and everyone is looking at them saying ‘maybe she has been raped’* (IDI, implementing partner, female, 30–40 years).

This was echoed during group discussions with PE officers, who described the challenges that AGYW face in accessing GBV services:

*There are lot of teenagers who have been raped and they never get any assistance. These teenagers would have to travel about 60 km to reach PVC centres. Where is this teenager going to get the money to get assistance?* (FGD, PE officer, female, 30–40 years).

Similarly, frontline researchers were apprehensive about following AHRI guidelines for referring study participants who experienced or were exposed to GBV because of the quality of the services. One research staff member observed: ‘… *we see very worrying acts and we think“you will report [this case], then what will happen next?”*’.

The requirement that research staff report GBV was a concern, not least because of the potential risks they faced by reporting violence, as explained by a frontline researcher:

*The guidelines state* *‘* *you might be required to go and testify in court* *’* *in the event the case may be taken to court. […] we also live in the same communities. So, if I go and testify against a family member in a child's rape case for instance, am I still going to be able to go back and be accepted in that community?* (FGD, frontline researcher, male, 20–30 years).

It is a legal offence to have sex below the age of 16 under any circumstance, and researchers are required to report these acts. Ethics committee members cautioned researchers to consider the context and the consequences of mandatory reporting if it was not done carefully. Indeed, many frontline researchers reported the practical implications of these tensions in their day-to-day activity, sometimes referring teenagers to health facilities for pregnancy screening or HIV testing, rather than pursuing mandatory reporting.

Researchers have clear procedures to report the case to the child protection lead at the AHRI, often leading to the involvement of a social worker and the department of social welfare. Despite this process, frontline researchers were often left facing difficult family tensions and dilemmas about the scope of their continued involvement given their relationship with the adolescent girl and her family. Sometimes family members turned a blind eye because the perpetrator was a breadwinner, and caregivers feared losing the source of their livelihood.

Reporting and referring these cases of GBV and child sexual abuse was further complicated by the tensions between the dual authority of the traditional and legal systems. This was a particular challenge for staff who are themselves from the local area. For example, the local practice requires reporting cases of abuse to traditional local leaders. The families of the perpetrator and the victims must negotiate the case with the local chief acting as mediator, often without any consideration of the law. These tensions compound the roles of frontline researchers who were reluctant to go against the local practices and authorities.

Support for younger adolescents (10–14 years) and the caregivers in managing the process of HIV disclosure was a challenge. While the caregivers perceived this as ‘protecting their children’, the AGYW (10–14 years) expressed hurt with the way this was managed:

*Our parents must tell us about our HIV status because it is painful, and it is depressing to learn about this way* (FGD, AGYW, female, 10–14 years).

The study referred AGYW who experienced emotional distress for counselling and support, and the IPs acknowledged that managing this process was a sensitive and complicated matter. They expressed anxiety about how this might affect relationships between adolescents and young people and their caregivers, as most caregivers tried to keep this a secret.

### How researchers responded to participants’ unmet needs

Frontline researchers reported being overwhelmed by the desire and moral obligation they felt to respond to participants’ needs. They expressed feelings of guilt and moral distress when they were unable to alleviate the suffering of study participants. As one frontline researcher said: ‘*…but what can you do?You cannot just ignore people's plight like that*’. A senior research team member expressed similar concerns: ‘*those things leave me a bit helpless and wish I could do more; […] when the staff come back from the field and they narrate all these sad stories*’.

Frontline researchers described frustration that whilst the nature of their research invariably drew out the challenges that their participants faced, they were unable to alleviate them. One frontline research team member commented: ‘*The community looks at us as people who are interested in them only when we want what we want; there's nothing we bring back to people as a form of support*’. Ultimately, they become a source of emotional support for participants.

The PE staff explained that they were particularly frustrated and distressed by being at the interface between the community and researchers and being unable to provide support:

*People share their stories and you wonder where do I even begin to help them? What makes it unbearable is the inability to go an extra mile to help them. It makes you emotional* (FGD, PE officer, male, 40–50 years).

Frontline researchers reported that debriefing sessions with their line managers and teams were important and helped them deal with emotional distress they often internalised. This also supported staff to become a source of social and emotional support for participants in distress.

### Lessons for responding to structural care needs within research

The findings from the interviews consistently showed that the responsibility to respond was borne by frontline staff, who often had the least power or resources to effect sustainable change. The study team developed a relationship with the service providers in the health and social system. Based on the Standard Operating Procedures and in communication with their supervisors, they referred AGYW/M to the nearest service based on need. However, findings showed that frontline researchers often felt that the ancillary support (particularly social support) they could refer to was not adequate. In addition, staff expressed concerns that referral protocols were inadequate to guide and support frontline staff to manage ancillary care needs. They, and participants, offered ideas for how to respond better to the broader needs. Most participants suggested that the AHRI align research with priorities identified by the community. They expressed the view that the Institute should put systems in place to address the health, social and economic needs of the community given the history of research in the community.

They suggested that the socio-economic characteristics of research participants provided ethical grounds for the research to ‘bring services to people’ and ‘do more’. One male frontline researcher commented:

*Why can't the institute allocate funds which will be dedicated to young people's issues we have raised, such as children who wish to further their studies? That would be a long-term investment in the communities we are working in male frontline researcher, 20-30 years*.

Study participants repeatedly focused on the community members’ precarious livelihoods, and ways to address poverty and bring about structural change. CAB members and PE officers reported that food vouchers were not adequate for families struggling to make ends meet. This was echoed during discussions with the caregivers:

*These* *[* *vouchers* *]* *are not sustainable. What if they give something that can be used long* *term …?* *The families are experiencing severe food insecurity* (FGD, caregiver, female, 30–40 years).

Ethics committee members echoed the view that research should benefit the participants and the community where research is taking place.

## Discussion

This in-depth case study of health research in a resource-constrained setting illustrates the complex unmet ancillary care needs of AGYW and the emotional challenges that face frontline researchers on a daily basis. The expectation that research studies in low-income settings must respond to participants’ health needs and prevent harm is embedded within the ethical, sociocultural, economic and political context in South Africa and internationally. Yet existing ethical frameworks and guidance do not always reflect the constraints of LMICs. Our data show that even in a study designed to be responsive to the broader, non-medical needs, researchers faced significant practical, moral and emotional challenges in determining the scope of their obligations to respond effectively to participant needs.

When conducting research with AGYW who have limited social capital, frontline researchers and staff often found themselves as critical sources of social support. This was most powerfully illustrated through researchers’ experiences in responding to GBV and the nexus between gender, youth and socioeconomic vulnerability.

Existing ancillary care guidance fails to fit the LMIC research context in several important ways, reconfirming calls for empirical research to inform frameworks that are more responsive to the LMIC context.^7^ Because early models of ancillary care emerged from high-income country settings,^18^ most guidelines continue to assume that the health service is publicly supported, or funded through insurance schemes; this may not be the case. A focus on primarily clinical duties of care overlooks other types of needs. Although the study showed potential for engaging government institutions including health, social welfare and law enforcement to deal with participants’ needs, these institutions themselves faced barriers, including limited resources and expertise, to providing quality services. Scholars of ancillary care ethics advocate for a paradigm shift from meeting individual participants’ needs.^19^ This requires that funders and research institutions must take more responsibility to identify institutional solutions and resources to improve the wider context, where research is conducted.

Also, because ancillary care emerged in clinical research and the clinician–patient relationship, existing ethical frameworks guiding ancillary care often focus on the duties of the individual researcher – especially clinician-researchers – rather than of a team, institution or sponsor. In a context of widespread social injustice, this means that individual researchers potentially bear a heavy burden. As the Georgetown working group has argued, this is both unfair and unsustainable – and, as we found, a significant source of moral distress for frontline staff.^7^ A focus on clinical researchers overlooks the role and obligations of non-clinical research team members at all levels, who encounter participants’ needs and feel they should respond.

By focusing on the intersection between the challenges and vulnerabilities experienced by research participants and how these manifest in the research encounter with a range of research staff and partners, we can identify critical points where questions around the nature and scope of duties of care need to be reconsidered. One critical point is the encounter between research staff and participants in situations of significant health or social needs. There is a clear expectation in the community that research ought to address the needs of participants and the community. Frontline research staff and those who bridge community and research – CAB members and PE officers – regularly and personally confront the tension between unmet expectations and the practical limits of how much research can do to respond to widespread, complex needs.

The researchers’ role becomes blurred as they receive requests for support that should have been provided by government or other service providers. It is not only a significant source of moral distress for researchers, but also risks undermining community expectations about what benefits research can provide. One potential solution that has been adopted in similar LMIC research centres is to build in ‘ethics reflections’ within research teams to offer a safe place to air concerns and to address practical dilemmas together, rather than leaving this for frontline staff to handle on their own.^20^^,^^21^ Another very important potential solution to this tension that the research team have adopted in response to this study is community-based participatory research. This places community members at the centre of the research process, provides fora for listening and responding to the community, as well as the infrastructure to support community leadership and advocacy development to generate relevant knowledge whilst supporting social change.^22^ Placing AGYW at the centre of the intervention development process using community-based participatory approaches to co-create contextualised interventions may alleviate some of the issues raised in this study.^20^

### Strengths and limitations

The study contributes to the literature on ancillary care by showing existing gaps in the guidelines and provision of ancillary care in resource-constrained settings where research participants may not only have health needs but also require economic and social support. We also demonstrated the emotional distress experienced by the researchers as they tried to balance participant expectations and ancillary care needs. One of the study limitations is that it lacks perspectives from the research institution leadership, funders and government institutions. Although there is a general understanding of structural challenges in the community, our findings are described from the lens of the CAB members and PE officers.

## Conclusion

The study highlighted existing gaps in meeting ancillary care needs and pointed to ways researchers may respond. Our findings show that participants’ needs required social and economic support, and that frontline researchers and implementing partners were not able to meet these needs. Recommendations were made for the institute to align research priorities with community needs.

### Supplementary data

Supplementary data are available at *International Health online* (http://inthealth.oxfordjournals.org/).

## Supplementary Material

ihaa045_Supplemental_File
