# Supplementary material for: Managing ancillary care in resource-constrained settings: Dilemmas faced by frontline HIV prevention researchers in a rural area in South Africa
Source: Int Health. 2020 Nov 7;12(6):543–50. doi: 10.1093/inthealth/ihaa045 (PMC7651306; doi:10.1093/inthealth/ihaa045)
Supplement: ihaa045_Supplemental_File [file ihaa045_supplemental_file.zip › Supplemntary_file_Interview_guides.docx]

**Supplementary files**

**Interview Guides**

**Adolescents/Young People – Enrolled in the HIV intervention programme**

Thank you for agreeing to participate in our REACH ethics study. I appreciate you taking time to speak with me. We are interested in finding out more about your thoughts, experiences, and views about participating in research studies and how this affects your daily life. We want to better understand how challenges or vulnerabilities in daily life are affected by being involved in research. And we also want to hear about things like coping strategies, choices, and benefits from research that you rely on to face challenges in daily living. We will talk for about an hour, but you can stop at any time. You do not need to answer any questions which you don’t want to answer. It is fine to answer only what you are comfortable talking about. There is no right or wrong answer to these questions. Your perspective really matters to us, so we really want to hear what you think and what your experiences have been.

Before we begin, I want to gather some basic background information that is helpful to us:

| Site name/ID |  |
| --- | --- |
| Interviewer |  |
| Participant Code |  |
| Date of IDI |  |
| Time of IDI | Time started: |
|  | Time ended: |
| Age of Participant |  |
| Gender of Participant |  |

**Section 1: Background and warm-up**

**The purpose is to build rapport and obtain background on the participants’ history, migration patterns (if any), household structure and livelihood.**

- 1. Can you tell me a little about yourself? What kinds of things do you like to do?
- E.g., do you like sports, what kind of music do you like?
  1. Do you have any brothers and sisters, cousins? (Get them talking about family, caregivers but be sensitive if talking with someone who has lost parents)
  2. Who are the main caregivers in your life?
  3. Do you also care for others in the family—younger brothers or sisters, nephews, nieces, neighbours?
  4. How long have you lived here/how you came to live here? Where are you from/where were you born?
  5. Are you currently in school? - level, what subjects do you really like, dislike?
  6. Are you working sometimes?
  7. What work do you or others in the house do?
- paid/unpaid, barter, gifts, grants, remittances
- nearby or outside community?
  1. What (other) unpaid work do you do in the house and community—helping around house, planting, selling things at market, fixing things, caring roles, volunteer?

**Section 2: Decision-making, sources of social support**

**The purpose is to explore participant’s (and household) agency and sources of support**

- 1. Do you get to make some decisions and choices about things in your life? What sorts of decisions? (what to do in free time, talking on phone, games, when to do schoolwork, job, choosing friends, other?)
  2. Who else makes decisions about things that affect your life? (caregivers, parents, grandparents, teachers, boyfriend/girlfriend, friends, other?)
  3. Can you tell me about your main sources of support or help from other people? (friends, family, elders in community, teacher, counsellor, other)

**Section 3: Challenges and coping strategies**

**The purpose is to understand possible background challenges, vulnerabilities and coping strategies**

- 1. Please think about challenges you have faced in life or might be facing now –social, economic, spiritual - what makes these situations difficult/challenging?
  2. How are you affected during difficult times (within the household, at school, in community)?
  3. What opportunities/support do you have that keeps you going? (community, school, church, support networks etc.)
  4. Now please think about your strength and courage when facing these difficult times. Where does your strength come from, what strategies do you employ to cope with difficulties?
  5. What do you think has been the biggest change in your life? How did you adapt to this change?

**[Possible visual, drawing activity here to facilitate a life story – key challenges and supports and people who have helped]**

Probe for narrative around:

1. Sources of vulnerabilities/challenges
2. Participants’ perspective on his/her position- empowered, vulnerable
3. What are the sources for these feelings (of empowerment and vulnerability)
4. Probe for sources of support, form of support and what this means for the participant.

**Section 3: Experiences participating in research**

**Thank you for sharing your thoughts and experiences on these things. Now I would like to ask some questions relating to being involved in a research study/HIV intervention programme and learn more about how this experience has affected your daily life, what you think has been helpful, not helpful.**

- 1. How did you decide to participate in research/HIV intervention programme? Was a caregiver also involved in the decision? Did you feel like you could decide to participate or not participate?
  2. How was your experience day-to-day, during study visits, etc.? (open ended, let them talk)
- What did you find helpful about participating in research/ HIV intervention programme? Probes: travel, food, other items, healthcare, support, met other kids, other benefits.
- What did you find less helpful about participating in research/ HIV intervention programme? Probes: costs, difficult travel, stigma, emotional distress, other challenges
  1. How did being involved in research affect other members in your family/household, like caregivers, brothers and sisters? (be open to positive or negative responses and probe if necessary)
  2. How was your experience with the informed consent and information in the study? Did things make sense? Was anything confusing about the information or what was happening?
  3. How was your experience with research staff members—the nurses, doctors, counsellors?
- Probes: without leading, positive and negative examples for how treated, helped, spoken with, stories of assistance, any difficult interactions.
  1. What is the general opinion towards the HIV intervention programme/research among other kids in the study, or in your community—do you hear people sometimes talking about HIV intervention programme? What do they say?
  2. Do you have any ideas or suggestions for how the researchers can make participation easier for you and your family?
  3. Is there anything you would like to add that you did not have a chance to say?

**Thank you very much for talking with me. It was really nice to meet you.**

**In-depth interview guide**

**Caregivers/guardians of children/adolescents involved in the HIV intervention programme/research**

Thank you for agreeing to participate in our REACH ethics study. I appreciate you taking time to speak with me. We are interested in finding out more about your thoughts, experiences, and views about having a child or family member participating in research studies and how this affects your daily life. We want to better understand how challenges or vulnerabilities in daily life are affected by being involved in research. And we also want to hear about things like coping strategies, choices, and benefits from research that you rely on to face challenges in daily living. We will talk for about an hour, but you can stop at any time. You do not need to answer any questions which you don’t want to answer. It is fine to answer only what you are comfortable talking about. There is no right or wrong answer to these questions. Your perspective really matters to us, so we really want to hear what you think and what your experiences have been.

Before we begin, I want to gather some basic background information that is helpful to us:

| Site name/ID |  |
| --- | --- |
| Interviewer |  |
| Participant Code |  |
| Date of IDI |  |
| Time of IDI | Time started: |
|  | Time ended: |
| Age of Participant |  |
| Gender of Participant |  |
| Relationship to the HIV intervention programme, study participant (father, mother, auntie, sibling, other) |  |
| Marital status | - Married - Widowed - Divorced - Separated - Abandoned - Live in partner - Single, relationship - Single, not current relationship |

**Section 1: Background - family life and livelihood**

**The purpose is to build rapport and obtain background on the participants’ history, migration patterns (if any), household structure and livelihood.**

- 1. Can you tell me a little about yourself and your family? Could you please tell me who stays here with you—parents, children, grandchildren?
  2. How long have you lived here/how you came to live here? Where are you from/where were you born?
  3. What work do you or others in the house do?
- paid/unpaid, barter, gifts, grants, remittances
- nearby or outside community?
  1. What (other) unpaid work do you do in the house and community—caring roles, volunteer?

**Section 2: Decision-making, sources of social support**

**The purpose is to explore participant’s (and household) agency and sources of support**

2.1 Who makes decisions in the house, most times?

- 1. What kinds of things do you make decisions about in the house? (child care/education, meals, shopping, healthcare, other) and outside the house? (at church, in the community, with friends)
  2. Can you tell me about your main sources of support or help from other people? (Friends, family, elders in community, teacher, other)

**Section 3: Challenges and coping strategies**

**The purpose is to understand possible background challenges, vulnerabilities and coping strategies**

- 1. Please think about challenges you have faced in life or might be facing now –social, economic, spiritual - what makes these situations difficult/challenging?
  2. Who is most affected during difficult times (within the household, at community)?
  3. What opportunities/support do you have that keeps you going? (community, school, church, support networks etc.)
  4. Now please think about your strength and courage when facing these difficult times. Where does your strength come from, what strategies do you employ to cope with difficulties?
  5. What do you think has been the biggest change in your life? How did you adapt to this change?

Probe for:

1. Sources of vulnerabilities/challenges
2. Participants’ perspective on his/her position- empowered, vulnerable
3. What are the sources for these feelings (of empowerment and vulnerability)
4. Probe for sources of support, form of support and what this means for the participant.

**Section 3: Experiences with family/child participating in research**

**Thank you for sharing your thoughts and experiences on these things. Now I would like to ask some questions relating to having a child/family member involved in a research study and learn more about how this experience has affected daily life challenges and sources of support, what you think has been helpful, not helpful.**

- 1. How did you decide to have your child participate in research/HIV intervention programme?
  2. How was your experience day-to-day, during study visits, etc.? (open ended, let them talk)
- What did you find helpful about participating in research/HIV intervention programme? Probes: travel, food, other items, healthcare, support, other benefits
- What did you find less helpful about participating in research/HIV intervention programme? Probes: costs, difficult travel, stigma, emotional distress, other challenges
  1. How did being involved in research or having a child/family member involved affect other members of the family or household? (be open to positive or negative responses and probe if necessary)
  2. How was your experience with the informed consent and information in the study?
  3. How was your experience with research staff members?
- Probes: without leading, positive and negative examples for how treated, helped, spoken with, stories of assistance, any difficult interactions.
  1. What is the general opinion towards HIV intervention programme /research in your community?
     1. Do you have any ideas or suggestions for how the researchers can make participation easier for you and your family?
  2. Is there anything you would like to add that you did not have a chance to say?

**Thank you very much for talking with me. We really value your insights and suggestions.**

**Individual Interview Guide***

**Investigators/ Researchers/ Fieldworkers**

***can be converted to group discussion as needed**

Thank you for agreeing to participate in our REACH study group discussion. I know you are very busy, so I really appreciate you taking time. As mentioned, this is a study on research ethics focusing on research involving vulnerable populations, so your perspective as an Investigator/ Researcher/ Fieldworker is very important. Specifically, we are interested in finding out more about your thoughts, experiences, and views about vulnerability, resilience and research in the communities and participants you work with. We are also interested to learn about your role/s and your suggestions for how research processes, support and ethics guidance can be improved. We will talk for about an hour, but you can stop at any time. You do not need to answer any questions which you don’t want to answer. It is fine to answer only what you are comfortable talking about. There is no right or wrong answer to these questions. Your perspective really matters to us, so we really want to hear what you think and what your experiences have been.

[**Note to interviewer:** in some questions, some small changes to the wording may be necessary depending whether you are speaking to an investigator or a researcher/fieldworker]

**Section 1: Background**

- 1. To start with, can you tell me a little about your professional background -what type of research do you do, where, and with which groups of people?
  2. Could you tell me more about your current role and responsibilities?
  3. How did you first get interested in research?
  4. What do you like most about your work, what do you find challenging about it?

**Section 2: Experiences of working with research and the community**

Thank you. Now I’d like to ask you a few questions about your observations of the participants and communities you have been working with, things you may directly observe, or things you may hear about from participants, colleagues, or out in the community:

- 1. Could you tell me about the kinds of challenges/ problems they face? Probe for challenges related to:
- Basic needs: health, nutrition, education, livelihoods, housing
- Identity and discrimination: gender, race, ethnicity, nationality, citizenship, religion, political affiliation, sexuality, (dis)ability
  1. Are there any people or households that are more affected/ at risk than others? In what way?
  2. Thinking about the challenges you have mentioned, in your observations how do they cope with these difficulties?

Probe for:

- What would you say are important sources of help or support?
- What are the personal, social or other strengths or abilities you have observed?

**Section 3: Ethical challenges along the research pathway:**

Ok, we’ve talked about the general background of the participants you work with. Now I’d like to hear about the different experiences - both positive and negative – that you, the research team, and the participants have during different stages of the research process.

- 1. Thinking about the typical research pathway below, do challenges arise at any of these points for the participants or for you as a researcher?

**For interviewer: If helpful, imagine or even draw the research pathway to facilitate probing**

| ***Engagement about research in the community*** | ***Participant Identification:***  ***Inclusion/exclusion decisions*** | ***Enrolment into study***  ***including compensation*** | ***Consent*** | ***Study procedures*** | ***Follow-up*** |
| --- | --- | --- | --- | --- | --- |

(WHAT CHALLENGES, WHO AFFECTED, HOW?)

- 1. A few follow-up questions specifically on consent:
     1. Who’s usually present?
     2. What normally happens? How much discussion? How many questions? What is the focus of discussions/questions? (if not much discussion, why not do you think?)
     3. How are the research elements generally understood by participants and families? Which elements are easiest to understand? Why? And hardest?
     4. Have you had many drop outs/rejections yet? What do you think contributed to this? Why do you think families agree? Is the decision difficult for them do you think?
     5. Do you think the process should be improved/changed? In what way?
  2. [Follow up on specific challenges emphasized by the researcher:] You mentioned [specific challenge] - How do you or your research team respond when this happens? What lessons have you learned? What in your experience are effective and ineffective ways of responding?
  3. What do you think are the main benefits of participation in your study/studies for the participants? And for their families? And what difficulties do they experience from participating in research?
  4. How about benefits for the health facilities/clinic/hospital? The broader local community here? And what would you say are the main disadvantages for them?

**Section 4: Perceived ethical obligations and scope of responsibilities:**

- 1. When you step back and reflect on some of the needs or challenges witnessed among the participants/patients/families in your studies, what do you feel is your personal obligation to respond? [Let them take time to think about this and talk] What makes you say that? Do you think anyone else bears responsibility for responding – if so, who and in what way, and why?
  2. Are there challenges or needs among your participants that you think fall outside the scope of your obligations as a researcher? What makes you say that?
  3. In your view, what role can research play in addressing some of the health, social, and economic needs you have witnessed amongst these communities?
- Do you think it is important to do research with these people and in these communities – if so, can you say why?
- When do you think it might be inappropriate to do research with certain people or in a certain area?
  1. In your own life, as a researcher, when you face some challenges or difficulties like the ones you have mentioned, what do you do? How do you cope? How do you overcome such situations? (Open ended, let them talk and only prompt if needed.)
  2. What are some of the main sources of support in your life and as a researcher?
  3. Overall, what do you think needs to change – what would make the most difference for research participants and researchers facing the challenges we’ve been discussing?
  4. Do you have specific suggestions for improving research ethics training or support for researchers who work in sometimes challenging contexts and with participants and families who might be considered vulnerable?
  5. Thank you. Is there anything else you would like to add, or any other thoughts about this general topic you would like to share with me? Thank you so much for taking time to talk with me. This has been very helpful. I wish you a good journey/day.

**Individual Interview Guide***

**Implementing Partners & Frontline Health workers**

***can be converted to group discussion as needed**

Thank you for agreeing to participate in our REACH study group discussion. I know you are very busy, so I really appreciate you taking time. As mentioned, this is a study on research ethics focusing on research involving vulnerable populations, so your perspective as an implementing partner in research is very important. Specifically, we are interested in finding out more about your thoughts, experiences, and views about vulnerability, resilience and research in the communities and participants you work with. We are also interested to learn about your role/s and your suggestions for how research processes, support and ethics guidance can be improved. We will talk for about an hour, but you can stop at any time. You do not need to answer any questions which you don’t want to answer. It is fine to answer only what you are comfortable talking about. There is no right or wrong answer to these questions. Your perspective really matters to us, so we really want to hear what you think and what your experiences have been.

**Section 1: Background**

- 1. To start with, can you tell me a little about your professional background -what type of work do you do, where, and with which groups of people?
  2. Could you tell me more about your current role and responsibilities?
  3. What do you like most about your work, what do you find challenging about it?

**Section 2: Experiences of working in the community**

Thank you. Now I’d like to ask you a few questions about the people, patients and communities you have been working with, things you may directly observe, or things you may hear about from patients, colleagues, or out in the community:

- 1. Could you tell me about the kinds of challenges/ problems they face? Probe for challenges related to:
     1. Basic needs: health, nutrition, education, livelihoods, housing
     2. Identity and discrimination: gender, race, ethnicity, nationality, citizenship, religion, political affiliation, sexuality, (dis)ability
  2. Are there any people or households that are more affected/ at risk than others? In what way?
  3. Thinking about the challenges you have mentioned, in your observations how do they cope with these difficulties? Probe for:
     1. What would you say are important sources of help or support?
     2. What are the personal, social or other strengths or abilities you have observed?

**Section 3: Experiences in delivering care and partnering with researchers/research projects**

Ok, we’ve talked about the general background of the people and communities you work with. Now I’d like to hear about the different experiences - both positive and negative – that you have encountered when partnering with research teams. We will use the HIV intervention programme as an example.

- 1. Can you describe your experience with the HIV intervention? What is your involvement?
  2. How do you work to integrate projects like this within your clinical/community context?
  3. What are some of the advantages or benefits of such partnerships?
  4. What are some of the challenges in your experience?
  5. Thinking about the typical research pathway below, do challenges arise at any of these points for the patients who participate in research studies or for you as a clinician/frontline health worker?

**For interviewer: If helpful, imagine or even draw the research pathway to facilitate probing**

| ***Engagement about research in the community*** | ***Participant Identification:***  ***Inclusion/exclusion decisions*** | ***Enrolment into study***  ***including compensation*** | ***Consent*** | ***Study procedures*** | ***Follow-up*** |
| --- | --- | --- | --- | --- | --- |

(WHAT CHALLENGES, WHO AFFECTED, HOW?)

- 1. A few follow-up questions specifically on consent:
     1. Who’s usually present?
     2. What normally happens? How much discussion? How many questions? What is the focus of discussions/questions? (if not much discussion, why not do you think?)
     3. How are the research elements generally understood by participants and families? Which elements are easiest to understand? Why? And hardest?
     4. Have you had many dropouts/rejections yet? What do you think contributed to this? Why do you think families agree? Is the decision difficult for them do you think?
     5. Do you think the process should be improved/changed? In what way?
  2. [Follow up on specific challenges emphasized:] You mentioned [specific challenge] - How do you or your research team respond when this happens? What lessons have you learned? What in your experience are effective and ineffective ways of responding?
  3. What do you think are the main benefits of participation in your study/studies for the participants? And for their families? And what difficulties do they experience from participating in research?
  4. How about benefits for the health facilities/clinic/hospital? The broader local community here? And what would you say are the main disadvantages for them?

**Section 4: Perceived ethical obligations and scope of responsibilities:**

- 1. When you step back and reflect on some of the needs or challenges witnessed among the participants/patients/families in your studies, what do you feel is your personal obligation to respond? [Let them take time to think about this and talk] What makes you say that? Do you think anyone else bears responsibility for responding – if so, who and in what way, and why?
  2. Are there challenges or needs among your participants that you think fall outside the scope of your obligations as a researcher? What makes you say that?
  3. In your view, what role can research play in addressing some of the health, social, and economic needs you have witnessed amongst these communities?
- Do you think it is important to do research with these people and in these communities – if so, can you say why?
- When do you think it might be inappropriate to do research with certain people or in a certain area?
  1. In your own life, as a clinician/health worker/researcher, when you face some challenges or difficulties like the ones you have mentioned, what do you do? How do you cope? How do you overcome such situations? (Open ended, let them talk and only prompt if needed.)
  2. What are some of the main sources of support in your life and work?
  3. Overall, what do you think needs to change – what would make the most difference for research participants and researchers facing the challenges we’ve been discussing?
  4. Do you have specific suggestions for improving research ethics training or support for researchers who work in sometimes challenging contexts and with participants and families who might be considered vulnerable?
  5. Thank you. We have covered a lot of territory – is there anything else you would like to add, or any other thoughts about this general topic you would like to share with me?

**Thank you so much for taking time to talk with me. This has been very helpful.**

**Key informant guide**

**Research ethics committee (REC) members**

Thank you for agreeing to participate in our REACH ethics study. I know you are all very busy so I really appreciate you taking time. As mentioned, this is a study on research ethics in research involving vulnerable populations, so your perspectives as ethics committee members are especially important. Specifically, we are interested in finding out more about your thoughts, experiences, and views about vulnerability, resilience and research in the surrounding communities. We are also interested to learn about your role/s on the REC and your suggestions for how research processes, support and ethics guidance can be improved. We will talk for about an hour and a half, but you can stop at any time. You do not need to answer any questions which you don’t want to answer. It is fine to answer only what you are comfortable talking about. There is no right or wrong answer to these questions. Your perspective really matters to us, so we really want to hear what you think and what your experiences have been.

**Section 1: Background**

1.1 Would you please talk about your role as part of the REC and how long you have served on the committee?

Group probes for:

- Would you say why you chose this role? (What is the process for becoming a REC member?)
- What do you like most about this position, what do you not like about this position?

**Section 2: Experiences with research**

- 1. Could you tell me more about the different kinds of research that the committee reviews?

Probe for:

- Who is involved in deciding what research takes place? Who has final say? (Explore power dynamics between REC, research teams, CAB, communities and research institute).
- What issues are you most concerned to check for when you’re reviewing research proposals?
- Do research studies ever take place here that you’re not happy with? Why/ in what way?
- Are there any types of research you would/ have turned down? If yes, why? [Probe for examples]
  1. What are the main ethical challenges that arise for your committee?
  2. Are there any issues that cause special concern for your group or as members?

**Section 3: Study populations-vulnerabilities, challenges, sources of support**

**Turning to the types of participants involved in research here, I’d like to ask a few questions about your impressions of the challenges and sources of support for people in this area who might be enrolled in research.**

- 1. Could you describe the groups of people who are usually asked to participate in research here?

Probe for:

- Are there any types/groups of people more likely to be excluded from research studies than others? Why?
- Are there any people that you think should be included but often aren’t? Why?
  1. How does this committee understand/characterize vulnerability? What does it mean to you in the research context?
  2. Are there any groups of research participant that you think need more protection than studies usually provide for? In what ways?
  3. Are there any groups that you think are overprotected? In what way?
  4. How does the ethics committee usually try to balance issues of overprotection and exploitation?
  5. Do current ethical guidelines do enough to help address issues of overprotection and exploitation?
  6. In your view, what is the role of ethics committees in being aware of or responding to vulnerabilities and challenges faced by people in the surrounding communities?
  7. In what ways do you find research helpful, and/not helpful?

Probe for:

- Ways that participating in research is beneficial and/ or difficult for people
- Do some people find participation in studies more beneficial or more difficult than others?
- Ways that research provides benefits and/or creates burdens for participants and communities

**Section 4: Support and recommendations**

- 1. In your view, is there anything that should be changed about the way research studies are planned or carried out here? How could they be improved, specifically where vulnerabilities and support are concerned?

Probe for:

- Ways that researchers can make participation easier for participants
- Ways to improve support for researchers who work with vulnerable populations
- Ways that ethical protections should be improved within guidelines/ review processes
  1. Thank you. We have covered a lot of territory – is there anything else you would like to add, or any other thoughts about this general topic you would like to share with me?

**Thank you so much for taking time to talk with me. This has been very helpful.**

**Group Discussion guide**

**CAB/ CEU members**

Thank you for agreeing to participate in our REACH ethics study group discussion. I know you are all very busy so I really appreciate you taking time. As mentioned, this is a study on research ethics in research involving vulnerable populations, so your perspectives CAB/CEU members are especially important. Specifically, we are interested in finding out more about your thoughts, experiences, and views about vulnerability, resilience and research in the surrounding communities. We are also interested to learn about your role/s on the CAB/CEU and your suggestions for how research processes, support and ethics guidance can be improved. We will talk for about an hour and a half, but you can stop at any time. You do not need to answer any questions which you don’t want to answer. It is fine to answer only what you are comfortable talking about. There is no right or wrong answer to these questions. Your perspective really matters to us, so we really want to hear what you think and what your experiences have been. Please be respectful of others and try to give everyone a chance to share experiences.

**Section 1: Background**

- 1. Going around, would you each please tell us about your role as part of the CAB/CEU and how long you have been in this role?

Group probes for:

- Would anyone care to say why you chose this role? (What is the process for becoming a CAB member?)
- What do you like most about this position, what do you not like about this position?

**Section 2: Challenges and sources of support in the community generally**

2.1 Could you tell me about the kinds of challenges/ problems facing people in this area? (e.g. related to: health, nutrition, education, livelihoods, housing, citizenship, access to services)

- 1. When people experience these problems, is there anything they do/ their households do to overcome or cope in that situation? What are some coping strategies you have witnessed?

Probe for:

- What are the different sources of support or help for people in the community?
- How easy or difficult is it for people to access these kinds of support?
  1. In your view, what is the role, if any, of the CAB/CEU in being aware of or responding to vulnerabilities and challenges faced by people in the surrounding communities?

**Section 3: Experiences with research and community**

***CAB/ CEU processes***

- 1. Could you tell me more about the different kinds of research that the CAB/ CEU engage with?

Probe for:

- Who is involved in deciding what research takes place? Who has final say? (Explore power dynamics between CAB, communities and research institute).
- What issues are you most concerned to check for when you’re reviewing research proposals?
- Do research studies ever take place here that you’re not happy with? Why/ in what way?
  1. What are the main challenges you experience as a CAB/CEU? As CAB/CEU members?

***Research participants and the community***

- 1. Could you describe the groups of people who are usually asked to participate in research here?

Probe for:

- Are there any types/groups of people more likely to be excluded from research studies than others? Why?
- Are there any people that you think should be included but often aren’t? Why?
  1. We are interested in your experiences and thoughts on research conducted in the community. In what ways do you find research helpful, and/not helpful?

Probe for:

- Ways that participating in research is beneficial and/ or difficult for people
- Do some people find participation in studies more beneficial or more difficult than others?
- Ways that research provides benefits and/or creates burdens for the community

**Section 4: Support and recommendations**

- 1. In your view, is there anything that should be changed about the way research studies are planned or carried out here? How could they be improved?

Probe for:

- Ways that researchers can make participation easier for participants
- Ways to improve support for researchers who work with vulnerable populations
- Ways that ethical protections should be improved within guidelines/ review processes
  1. Thank you. We have covered a lot of territory – is there anything else you would like to add, or any other thoughts about this general topic you would like to share with me?

**Thank you so much for taking time to talk with me. This has been very helpful.**

**Group Discussion guide***

**Community Members, Activists, Representatives**

***can be converted to individual interview as needed**

Thank you for agreeing to participate in our REACH ethics study group discussion. I know you are all very busy, so I really appreciate you taking time to speak with me. REACH is a study on research ethics. We are interested in finding out more about your thoughts, experiences, and views about vulnerability, resilience and social support in the community and how you think research engages these issues. We are also interested in your suggestions for how research processes can be improved. We will talk for about an hour and a half, but you can stop at any time. You do not need to answer any questions which you don’t want to answer. It is fine to answer only what you are comfortable talking about. There is no right or wrong answer to these questions. Your perspective really matters to us, so we really want to hear what you think and what your experiences have been. Please be respectful of others and try to give everyone a chance to share experiences.

**Section 1: Background**

- 1. Going around, would you each please tell us something about your role in the community, or what you do?

**Section 2: Challenges and sources of support in the community generally**

- 1. Could you tell me about the kinds of challenges/ problems facing people in this area? (e.g. related to: health, nutrition, education, livelihoods, housing, citizenship, access to services)
  2. When people experience these problems, is there anything they do/ their households do to overcome or cope in that situation? What are some coping strategies you have witnessed?

Probe for:

- What are the different sources of support or help for people in the community?
- How easy or difficult is it for people to access these kinds of support?
  1. In your view, who or what institutions can or should respond to vulnerabilities and challenges faced by people in the surrounding communities?

**Section 3: Experiences with research and community**

- 1. Are you aware of any research or types of research studies that have been happening in your communities? Examples?
  2. Who usually decides what research takes place out in the community? Who decides what types of issues are addressed?
  3. Going back to our discussion of challenges and sources of support in the community, in your view, does research or do research institutions have any role to play in responding to these issues? Why or why not, in what way, etc.
  4. We are interested in your experiences and thoughts on research conducted in the community. Can you think of examples where research has been helpful or brought benefits to the participants/community? What kinds of benefits?
  5. Can you think of examples or ways that research has been difficult for people or created challenges for them?
  6. Have any research studies ever taken place here that people in the community were not happy with?
- Why/ in what way—what specific concerns were raised?
- Any suggestions for how those concerns could have been addressed?
  1. Could you describe the groups of people who are usually asked to participate in research in your community?

Probe for:

- Are there any types/groups of people more likely to be excluded from research studies than others? Why?
- Are there any people that you think should be included but often aren’t? Why?

**Section 4: Support and recommendations**

- 1. In your view, is there anything that should be changed about the way research studies are planned or carried out here? How could they be improved?

Probe for:

- Ways that researchers can make participation easier for participants
- Ways to improve support for researchers who work with vulnerable populations
- Ways that ethical protections should be improved within guidelines/ review processes
  1. Thank you. We have covered a lot of territory – is there anything else you would like to add, or any other thoughts about this general topic you would like to share with me?

**Thank you so much for taking time to talk with me. This has been very helpful**.
