# Supplementary material for: Managing ancillary care in resource-constrained settings: Dilemmas faced by frontline HIV prevention researchers in a rural area in South Africa
Source: Int Health. 2020 Nov 7;12(6):543–50. doi: 10.1093/inthealth/ihaa045 (PMC7651306; doi:10.1093/inthealth/ihaa045)
Supplement: ihaa045_Supplemental_File [file ihaa045_supplemental_file.zip › Supplementary_Figure1_Framework_for_HIV_core_packages.pptx]

## Slide 1
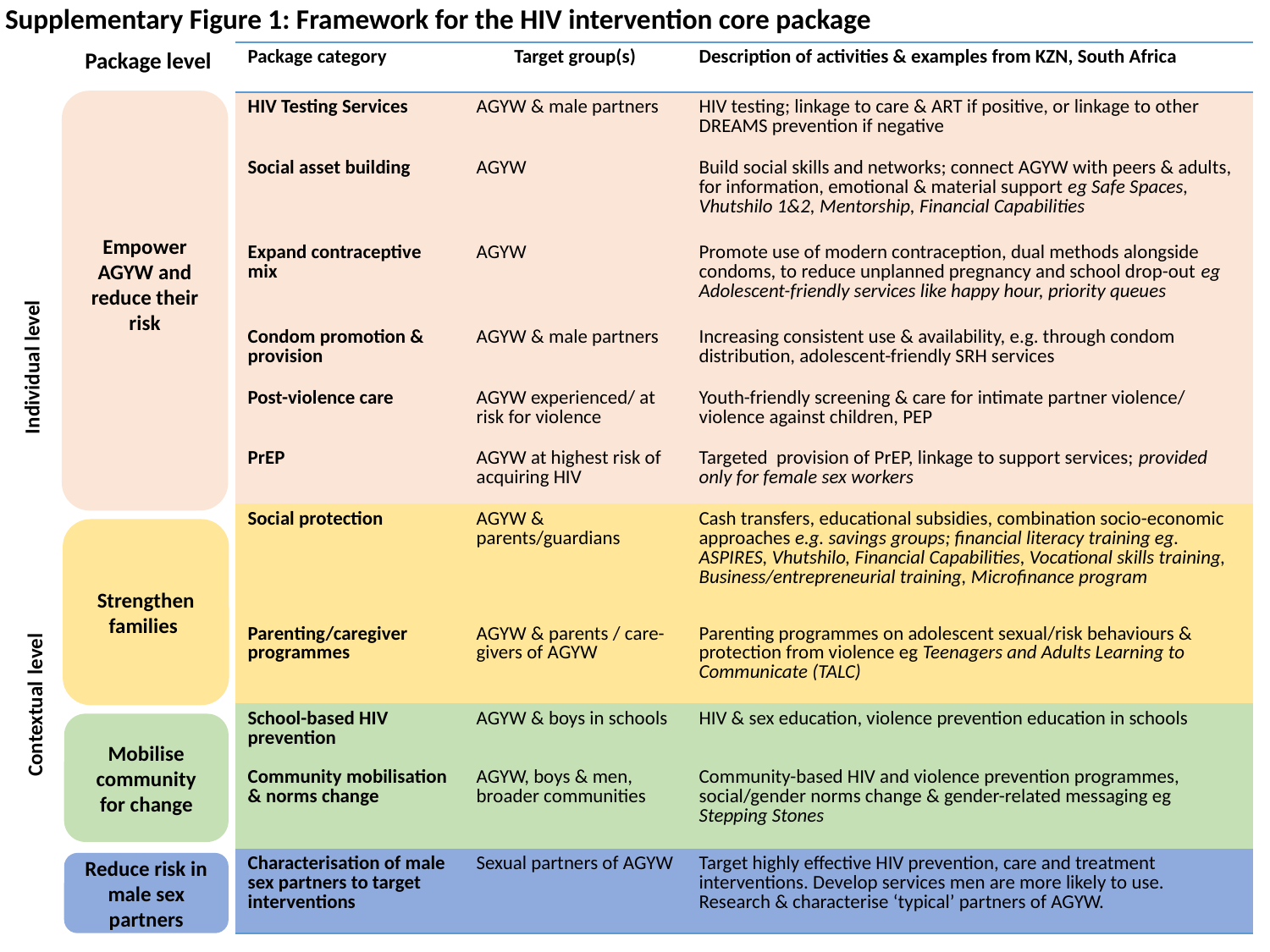

Supplementary Figure 1: Framework for the HIV intervention core package
Package level
| Package category | Target group(s) | Description of activities & examples from KZN, South Africa |
| --- | --- | --- |
| HIV Testing Services | AGYW & male partners | HIV testing; linkage to care & ART if positive, or linkage to other DREAMS prevention if negative |
| Social asset building | AGYW | Build social skills and networks; connect AGYW with peers & adults, for information, emotional & material support eg Safe Spaces, Vhutshilo 1&2, Mentorship, Financial Capabilities |
| Expand contraceptive mix | AGYW | Promote use of modern contraception, dual methods alongside condoms, to reduce unplanned pregnancy and school drop-out eg Adolescent-friendly services like happy hour, priority queues |
| Condom promotion & provision | AGYW & male partners | Increasing consistent use & availability, e.g. through condom distribution, adolescent-friendly SRH services |
| Post-violence care | AGYW experienced/ at risk for violence | Youth-friendly screening & care for intimate partner violence/ violence against children, PEP |
| PrEP | AGYW at highest risk of acquiring HIV | Targeted provision of PrEP, linkage to support services; provided only for female sex workers |
| Social protection | AGYW & parents/guardians | Cash transfers, educational subsidies, combination socio-economic approaches e.g. savings groups; financial literacy training eg. ASPIRES, Vhutshilo, Financial Capabilities, Vocational skills training, Business/entrepreneurial training, Microfinance program |
| Parenting/caregiver programmes | AGYW & parents / care-givers of AGYW | Parenting programmes on adolescent sexual/risk behaviours & protection from violence eg Teenagers and Adults Learning to Communicate (TALC) |
| School-based HIV prevention | AGYW & boys in schools | HIV & sex education, violence prevention education in schools |
| Community mobilisation & norms change | AGYW, boys & men, broader communities | Community-based HIV and violence prevention programmes, social/gender norms change & gender-related messaging eg Stepping Stones |
| Characterisation of male sex partners to target interventions | Sexual partners of AGYW | Target highly effective HIV prevention, care and treatment interventions. Develop services men are more likely to use. Research & characterise ‘typical’ partners of AGYW. |
Empower AGYW and reduce their risk
Individual level
Strengthen families
Contextual level
Mobilise community for change
Reduce risk in male sex partners
